# Supplementary material for: Complexome profiling of the Chlamydomonas psb28 mutant reveals TEF5 as an early PSII assembly factor
Source: Plant Cell. 2025 Mar 17;37(6):koaf055. doi: 10.1093/plcell/koaf055 (PMC12164590; doi:10.1093/plcell/koaf055)
Supplement: koaf055_Supplementary_Data [file koaf055_supplementary_data.zip › psb28_interactive.zip!/plots/intensity/qualityPlots/peptides/PETM_Cre12.g546150.t1.2.html]

### Cre12.g546150.t1.2

Trivial name: PETM  
SeqCoverage [median]: 10.10  
Mass: 10.00 kDa  
  
 

| Sequence | MissedCleavage | Mass [Da] | wtIntensity | muIntensity | Score |
| --- | --- | --- | --- | --- | --- |
| VESLVEEGK | 0 | 988.51 | 814.92 | 3897.05 | 122.52 |
| VESLVEEGKI | 1 | 1101.59 | 59521.07 | 109303.39 | 308.48 |
|  |  |  | 60336.0 | 113200.4 |  |
